# The Role of Scanning Electron Microscopy in the Evaluation of Conjunctival Microvilli as an Early Biomarker of Ocular Surface Health: A Literature Review

**DOI:** 10.3390/jcm13247569

**Published:** 2024-12-12

**Authors:** Mario Troisi, Salvatore Del Prete, Salvatore Troisi, Antonio Del Prete, Carlo Bellucci, Daniela Marasco, Ciro Costagliola

**Affiliations:** 1 Eye Clinic, Department of Neurosciences, Reproductive and Odontostomatological Sciences, Federico II University, 80131 Naples, Italy; antonio.delprete@unina.it (A.D.P.); ciro.costagliola@unina.it (C.C.); 2 Service Biotech s.r.l., 80121 Naples, Italy; saldelp@servicebiotech.com (S.D.P.); danielamarasco.servicebiotech@gmail.com (D.M.); 3 Ophthalmologic Unit, Salerno Hospital University, 84100 Salerno, Italy; 4 Ophthalmology Unit, Department of Medicine and Surgery, University Hospital of Parma, 43126 Parma, Italy; carlo.bellucci@unipr.it

**Keywords:** conjunctival microvilli, impression cytology, scanning electron microscopy, ocular surface disease, epithelial surface, ocular side effects

## Abstract

Microvilli are bristle-like protuberances of the plasma membrane, which express the vitality of mucous and epithelial cells; their alteration indicates a condition of cellular suffering in a predictive sense, making it possible to establish how much an inflammatory state or toxic conditions affect cellular functionality. In this article, the authors evaluate the applications of scanning electron microscopy (SEM) examination to impression cytology (IC) of the bulbar conjunctiva for the assessment of microvillar alteration as an early ultrastructural indicator of ocular surface health. This method offers several advantages, starting with its simplicity: it involves the non-invasive application of a strip of bibulous paper to the bulbar or tarsal conjunctiva. Unlike conjunctival or corneal biopsies, which are surgical procedures, this technique is far less invasive and more comfortable for the patient. It also provides a more clinically relevant in vivo assessment compared to studies on cultured cell lines, which are mostly limited to scientific research and may not accurately reflect real-world conditions. This makes it an effective, repeatable, and patient-friendly option for detecting early pathological alterations of the ocular surface. It also represents a useful tool for evaluating the efficacy of topical drugs and the toxic effects of external factors and ophthalmic or systemic diseases. Finally, it allows for obtaining accessory information relating to goblet cells, the presence of inflammatory infiltrate, or any pathogens.

## 1. Introduction

The conjunctiva is a thin translucent mucous membrane derived from surface ectoderm during embryologic development, constituted of two layers, the epithelium and the substantia propria. It covers the posterior surfaces of the two eyelids and is reflected forward on the anterior surface of the ocular globe [1,2,3,4]. The conjunctival epithelium is squamous type, with a deep layer formed of cylindrical cells, which adhere to a thin basement membrane trough hemidesmosomes, covered by 2–5 layers of polyhedral cells, which appear flattened on the surface [5,6]. The apical epithelial cells connect with each other by tight junctions that play a role in the permeability barrier, and they are covered by tear film. The stroma attaches to the underlying sclera and contains structural and cellular elements, including nerves, lymphatics, and blood vessels. The conjunctiva contributes to the formation of the tear film by way of secreting substantial electrolytes, fluid, and mucins [1,5,7,8].

On the conjunctival surface, there are goblet cells interspersed among epithelium, which are rounded cells, with flattened nuclei; they are more numerous in the caruncle, semilunar fold and fornix areas, while they are essentially absent from the limbal conjunctiva [9,10,11,12,13]. The cytoplasm of goblet cells is filled with string-like collections of mucin released on the surface of the conjunctiva [14]. Mucins secreted by goblet cells are a fundamental component of tear fluid, as they exhibit viscoelastic properties and interact with the external terminations of membrane glycoproteins of epithelial cells (glycocalyx) [9,15,16,17].

The speciments of normal epithelial conjunctivae surfaces, examined by SEM, show a characteristic uniform distribution of microvilli; less than 5% of clinically healthy conjunctival cells have non-uniform microvillar patterns [18].

Microvilli are tiny, finger-like projections on the surface of epithelial apical cells’ plasma membrane, which express the vitality of mucous and epithelial cells; they increase the surface area for absorption and secretion and play a role in maintaining the health of the ocular surface. Their function is closely related to the stability of the tear fluid: the deep layer of the tear film binds to the microvilli of the epithelial cells [19,20,21,22]; furthermore, transmembrane mucins are anchored to the ends of the epithelial surface, thus ensuring adequate wettability [23]. The microvilli and glycocalyx of the conjunctival epithelium provide support to the tears by determining the formation of a thin layer and preventing their fall and escape from the ocular surface by gravity [24]. In fact, the maintenance of tear film in normal conditions is dependent on mucus layer integrity and the presence and distribution of conjunctival epithelial cell microvilli [25].

Microvilli alteration indicates a condition of cellular suffering; therefore, their degree of alteration is significant for establishing the consequences of an inflammatory state or toxic conditions on cellular functionality [23,26,27].

Goblet cells may also have microvilli. An electron microscope study of the surface of the conjunctiva revealed that it is possible to classify the goblet cells into small, mature, and hypermature cell types [28]. The small goblet cells have flat surfaces, elliptical contours and some short microvilli. Mature cells are more globose and taller than the surrounding tissue, with rare microvilli on their surface; hypermature goblet cells are globose, but lack microvilli [13].

Microvilli assessment of epithelial cells is easily performed by scanning electron microscopy (SEM), a powerful imaging technique that provides high-resolution, three-dimensional images of the surface of specimens [29]. It uses a focused electron beam to scan the specimen and detect secondary or backscattered electrons. SEM provides detailed images of the size, density, and surface morphology of microvilli.

SEM has been employed in ocular surface research since the 1980s, offering unprecedented insights into conjunctival microstructures. Early applications demonstrated its ability to visualize epithelial features, such as microvilli density and morphology, which are indicative of ocular surface health. These studies laid the foundation for its use in diagnosing dry eye syndrome and other conditions characterized by epithelial damage. As SEM techniques evolved, improvements in resolution and sample preparation enabled the detailed visualization of ultrastructural changes, making SEM a critical tool in ocular surface diagnostics [5,9,14].

This assessment method has previously been performed primarily on conjunctival biopsy specimens, which are obtained by surgical procedure; more recently, in vivo impression cytology has been introduced, a non-invasive and easily reproducible technique that allows the superficial cell layer to be removed by simply placing a strip of a specific paper on the conjunctiva [30,31,32,33]. This procedure makes non-invasive performance of the examination possible in normal clinical practice.

The purpose of this work is to present a conceptual review of the application of scanning electron microscopy (SEM) in the evaluation of conjunctival microvilli. It encompasses an analysis of studies conducted over time, ranging from the earliest experiences using biopsy samples to the most recent studies employing impression cytology. The review highlights the primary clinical applications of SEM in diagnosing and managing ocular surface diseases and explores future perspectives in this evolving field.

## 2. SEM Applied to Biopsy

### 2.1. Biopsy Studies in Animals

The first scanning electron microscopy studies of the conjunctiva were performed on biopsy specimens; because SEM is a rather invasive procedure, it was initially performed on animals. Studies of rabbit conjunctiva showed a uniform pattern of fine, finger-like cytoplasmic protuberances (microvilli) covering the polygonal cells. Full-thickness holes of variable size were also detected, interpreted as the result of a sequential pattern leading to cell destruction and exfoliation. Interspersed between these polygonal cells, which were classified as light and dark, were goblet cells representing various stages of their maturation [14,19]. SEM examination of guinea pigs demonstrated corneal and conjunctival surfaces covered by microvilli and the presence of branching membranous folds up to 2 microns long, called microplicae, which increase the surface area of the superficial corneal epithelial cells, improving tear film stability. These surface projections were shortest (150 nm) on the central cornea and became progressively longer (about 300 nm) on the tarsal and fornical conjunctiva. A filamentous cellular lining (glycocalyx) was present on the microvilli and microplicae, extending about 300 nm from the tips and lateral surfaces of the microvilli and microplicae. The microvilli, microplicae, and glycocalyx provide the framework that supports and binds a complex of interrelated factors, including tears, mucus, and immunoglobulins, that have the common function of protecting the eye [34].

The surface of the conjunctiva has also been studied in goats, sheep, and cattle. At high magnification, the superficial cells of the palpebral and bulbar conjunctiva are covered by microplicae. In the conjunctival fornix, the microvilli protrude onto the surface cells [35].

The effects of administration of tear substitutes on normal mouse conjunctival epithelium were also studied. The results strongly suggest that tear substitutes can induce specific changes in the conjunctival epithelium with particular regard to the structure and ultrastructure of goblet cells [36]. Furthermore, a protective effect of vitamin A on the ocular surface of rats has been demonstrated by SEM evaluation on biopsies [37].

### 2.2. Biopsy Studies in Humans

Animal studies have been very useful in understanding the structure of the ocular surface, the interactions of cells with the tear film, and the effects of external factors on epithelial and goblet cells. These acquisitions paved the way for biopsy studies of the human conjunctiva. The conjunctival and corneal surfaces of human cells are normally covered with microplicae and microridges. These surface furnishings, the intercellular junctions, orifices of tear ducts and mucous glands in the conjunctiva, and certain hole-like structures on the epithelial cells could be clearly seen by SEM in three dimensions and provided useful information on the health of the ocular surface [38].

SEM has been applied to conjunctival biopsies of dry eye patients and has revealed the fine structure of cellular and nuclear changes (nuclear segmentation, abnormal chromatin distribution, reduction or loss of goblet-cells, signs of squamous metaplasia) and marked reduction in microvilli [39,40].

Changes in goblet cells just before and during secretion were also observed, including changes in surface cell diameter, evagination of the apical surface of the cell membrane, decreased number of microvilli, and alteration in the arrangement and morphological characteristics of the microvilli. Changes seen by light and transmission electron microscopy correlated with those observed by scanning electron microscopy [41].

**Figure 1:**
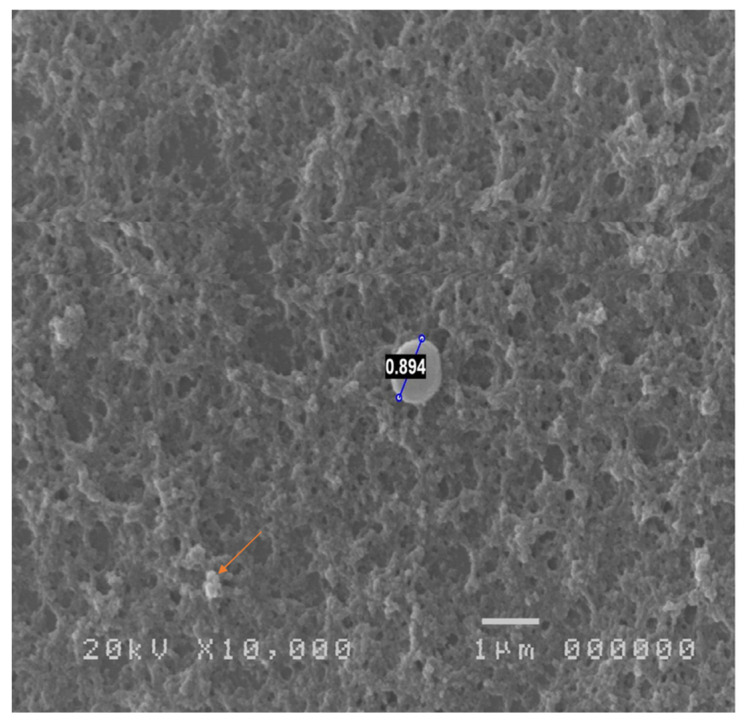
Mild alteration of microvillar surface in contact lens wearer; thickening of mucus (orange arrow) and presence of coccus in center of field are detected.

Biopsy specimens of the superior tarsal conjunctiva of asymptomatic contact lens wearers and patients with contact lens-associated giant papillary conjunctivitis (GPC), examined by SEM, showed branching from the side of a straight tubular microvillus, forming an acute angle with the main process. The most common branching pattern was bifurcated; occasionally, both primary and secondary bifurcations on the same microvillus were observed, as well as greater polymorphism, which was related to the degree of alteration [42] (Figure 1).

Conjunctival tissue samples from patients with Sjögren’s Syndrome (SS), obtained by bulbar conjunctival biopsy and examined by transmission electron microscopy, showed a significantly lower mean number of microvilli compared to the control group with healthy conjunctiva; furthermore, the height of microvilli and the height-to-width ratio in the conjunctival epithelium in the SS group were significantly lower [43].

In another study, the conjunctival surfaces of ten patients with active ocular cicatricial pemphigoid, three patients with drug-controlled ocular cicatricial pemphigoid, and six patients with normal conjunctivae were evaluated via SEM. A homogeneous, thick granular sheet of amorphous mucin-like material covering extensive areas of the conjunctiva was observed in eight of ten patients with active ocular cicatricial pemphigoid; this sheet of amorphous material was absent in drug-controlled ocular cicatricial pemphigoid and normal conjunctival specimens. This observation is compatible with clinical observations of thick mucus strands in the inferior fornix of patients with active ocular cicatricial pemphigoid [44].

Studies conducted on humans corroborate the observations made on animals, since the structures and alterations observed in animals are substantially comparable to human results; they have allowed us to establish a correlation between ultrastructural alterations, in particular the reduction in microvilli, and clinical signs of dryness and other ocular pathologies. The loss of microvilli is associated in all studies with an instability of the tear film, probably due to a reduced capacity of adhesion and stratification of the tear fluid on the surface of the epithelium.

## 3. Cell Culture Tests

Primary cultures of human epithelial cells from normal conjunctiva were developed and characterized to determine whether they retained epithelial characteristics. Conjunctival explants were obtained from the superior fornix of healthy donors and cultured in supplemented DMEM/F-12 medium. Primary cultures showed typical characteristics of conjunctival epithelium under light microscopy (polygonal morphology, intima cohesion, mucin production), TEM (abundant desmosomes, keratin bundles, granules, microvilli), SEM (polygonal shape, microvilli, intima cohesion), and immunocytochemistry (positivity for epidermal growth factor receptor, desmosomal proteins, and cytokeratins). The studies confirmed that primary cultures grown from normal human conjunctiva maintained epithelial characteristics in vitro. This in vitro system was therefore proposed for pathophysiological and toxicological studies [45].

Human conjunctival cell lines were also employed to compare the toxicity of a short-term application of timolol with benzalkonium chloride (T-BAC+) and timolol without preservatives (T-BAC−). T-BAC+ induced a rapid decrease in cell viability of 40% immediately after treatment and 85% 24 h later, compared to milder and more transient effects of timolol-BAC− [46]. This study proves the in vitro toxic effects of antiglaucoma drugs, which are more serious and, in part, made irreversible by products preserved with BAC.

In vitro evaluation of microvilli was also used to investigate the cytotoxic effect of topical ocular allergic agents with H1 receptor antagonism and inhibition of histamine release from mast cells on conjunctival cells in rabbit culture in vitro. The morphological changes determined on conjunctival cells have been correlated with pathological changes in some biochemical parameters, such as the loss of the enzyme lactate dehydrogenase (LDH) [47].

Furthermore, experimental dry eye models have been developed using in vitro reconstructed human corneal epithelium (HCE), on which changes similar to those determined by dryness are induced using particular culture conditions. With this method, the effects of various tear substitutes on the condition of induced dry eyes were tested. This in vitro dry eye HCE model can be satisfactorily used for the preliminary evaluation of the protective activity of artificial tears [48].

The study of the ultrastructural characteristics of cultured corneal and conjunctival epithelial cells (HCECs) has allowed for evaluating alterations induced by cigarette smoke (CS) pollution, which are correlated with clinical effects observed in vivo, to clarify the probable pathogenetic mechanism [49].

The availability of in vitro models of cell lines makes it possible to study different aspects of cellular function independently from the environmental context and the physiological or pathological conditions of the organism. The use of in vitro cultures also allows for testing a large number of substances in a short time and verifying their effects on countless biochemical and morphological parameters. However, the results of in vitro models must generally be verified with preclinical studies as they are not always generalizable in real life.

## 4. SEM Applied to Impression Citology (ICSEM)

Impression cytology is widely used as a non-invasive alternative to full-thickness biopsy to obtain epithelial cells from the ocular surface [50]. Some recent studies used impression cytology with SEM to study the conjunctival surface of animal [51] and human eyes [49].

To facilitate the scanning electron microscopy (SEM) analysis, conjunctival epithelium specimens are collected using the impression cytology technique, a minimally invasive method for sampling the ocular surface. This process involves applying a fragment of cellulose acetate to the upper-temporal bulbar conjunctiva for 3–4 s without the use of anesthetic. The collected specimens are then transferred onto a glass slide by pressing the cellulose acetate fragment against the slide for 30 s to ensure sample adherence. For SEM preparation, the epithelial samples undergo fixation in 3% glutaraldehyde dissolved in 0.065 M phosphate buffer (pH 7.4) for two hours at room temperature. Following fixation, the specimens are washed three times in phosphate buffer (30 min per wash) and then immersed in 1% osmium tetroxide (OsO_4_) in the same buffer for 30 min. The dehydration process is carried out through a graded ethanol series, and the samples are subsequently dried using critical-point drying with a CO_2_ liquid Bemar SPC 1500 apparatus (Bomar Co., Tacoma, WA, USA). The dried specimens are mounted on aluminum stubs using silver-conducting paint, coated with a 20-nanometer layer of gold using a sputter-coating technique, and observed under SEM. This comprehensive preparation ensures high-resolution imaging for detailed analysis of the conjunctival epithelium’s ultrastructural features [26].

Conjunctival microvilli are microscopic cellular membrane protrusions on apical epithelial cells, which increase the surface area available for tear adherence [52]. Normal microvilli are uniformly distributed and well formed. Abnormalities might include changes in size, shape, or density, indicating potential pathological conditions.

**Figure 2:**
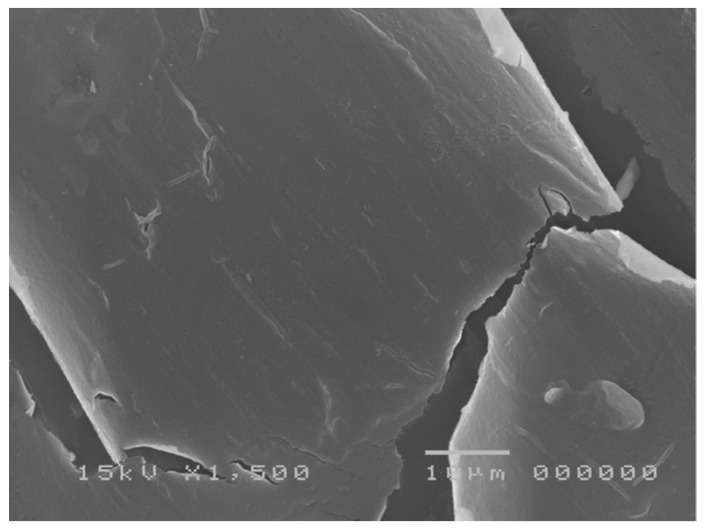
Severe dry eye dysfunction in patient with Sjogren’s syndrome: presence of epithelial conjunctival cell without microvilli; alteration of tight junctions and formation of fissures between the cells.

SEM studies have recently been performed on conjunctival impression cytology (ICSEM) specimens in patients with tear film abnormalities. Pathological alterations of the structure of microvilli affect the tear film stability and, conversely, dysfunctions of tear film composition can lead to a suffering epithelium (dry eye syndrome) [53,54] (Figure 2).

In a study by Cennamo et al. on forty-five patients with dry eye and fifteen asymptomatic subjects (control group) subjected to ICSEM examination, microvilli alterations were highly correlated with the subjective sensation of dry eye (Spearman correlation coefficient, 796; *p* < 0.01) and with alterations in clinical tests (tear breakup time, Schirmer test, and Ferning test); furthermore, the reduction or absence of microvilli revealed incipient epithelial damage before the appearance of alterations in the nucleus and cytoplasm of epithelial cells detected by light microscopy [55]. The strong limitation of Raman analysis is that the Raman microscope analyzes the proinflammatory or structural molecules present in the cell; the analysis, which is a laser spectrometry, offers complex and not immediately understandable results, unlike the SEM image, which is easily understandable as it visualizes the tissue as we see it. In practice, the structural alterations with SEM are immediately visible and the diagnosis in this sense is simpler and quicker.

A classification of the alterations of the microvillar surface of the conjunctiva in 5 degrees (from grade 0 to grade 4), subsequently used in other works, was also proposed; this is a preset scale based on the state of the surface, as well as the presence, distribution, and morphology of the microvilli [55] (Table 1).

**Figure 3:**
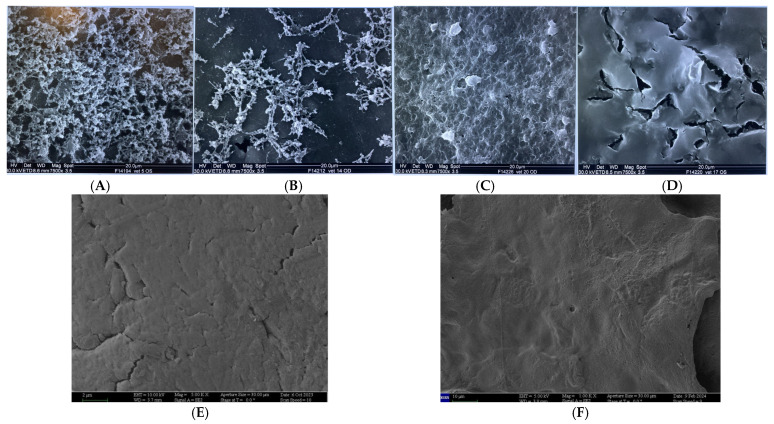
Grade 1 of Del Prete’s scale: low microvillar distribution, with microvillar structure not completely arborescent (**A**); grade 2 of Del Prete’s scale: microvillar distribution on spot and presence of pseudovilli (**B**); grade 3 alteration: severe impairment of the ocular surface, with significant reduction in microvilli, and presence of smooth areas and pseudomicrovilli (**C**); grade 4: microvillar absence with moon-like surface (**D**–**F**).

Figure 3A–D show SEM images relating to alterations of microvilli of grades 1 to 4 according to Del Prete’s classification (Figure 3A–F).

Scanning electron microscopy has also been applied to impression cytology to evaluate conjunctival damage in patients undergoing topical therapy for glaucoma. The conjunctival epithelium was evaluated with the ferning test (FT), impression cytology with light microscopy (ICOM), and impression cytology with scanning electron microscopy (ICSEM). Treatment duration was significantly associated with a reduction in microvilli count on ICSEM, but not with FT grades or ICOM. From this study, it emerges that the reduction in microvilli could be the first sign of cellular damage related to chronic glaucoma therapy [56].

In recent studies by Troisi M et al., SEM evaluation of the ultrastructural effects on conjunctival epithelial cells of a new multiple-action artificial tear containing cross-linked hyaluronic acid, cationic liposomes, and trehalose was carried out; it was also possible to identify the microscopic structure of the tested product and this allowed for verifying its persistence on the ocular surface 12 h after the last administration, presumably due to the mucoadhesive properties of the eye drops and the presence of cationic liposomes. This tear substitute has also proven to be very effective in restoring the damaged microvillar surface, thanks to the presence of cross-linked hyaluronic acid and trehalose. All treated subjects showed an improvement in the state of the microvilli after thirty days of twice-daily treatment, with a complete normalization of the epithelial surface in two of the three treated subjects and a significant increase in microvilli in all cases; it was also observed that the product penetrated between the microvilli, reinforcing them and restoring their arborescent structure, or covered the cell surface like a blanket, promoting the growth of the microvilli to which it binds strongly [24,57]. Cytological studies via SEM are able to demonstrate the trophic and protective effects of the substances tested precisely on the basis of their ability to stimulate the formation of normal microvilli in conditions of an arid or damaged ocular surface. The possibility of verifying the persistence of a product on the ocular surface after identifying its structure in electron microscopy is also interesting. The test therefore allows for establishing the residence time of a product on the ocular surface, after instillation in the form of drops.

**Figure 4:**
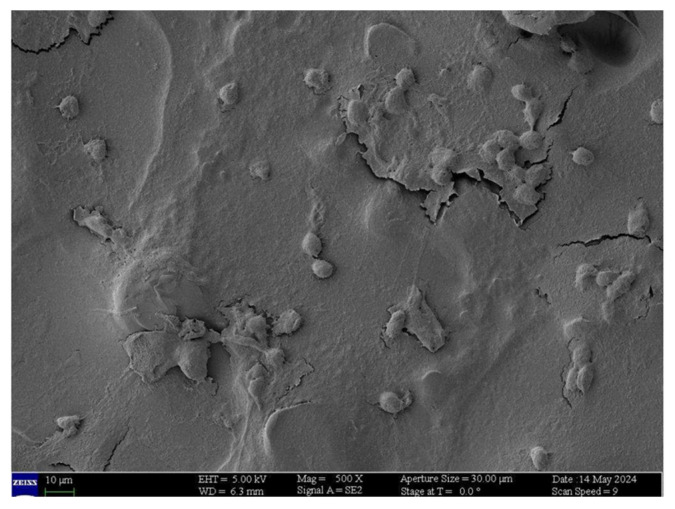
Leukocytic infiltrate in viral keratoconjunctivitis.

**Figure 5:**
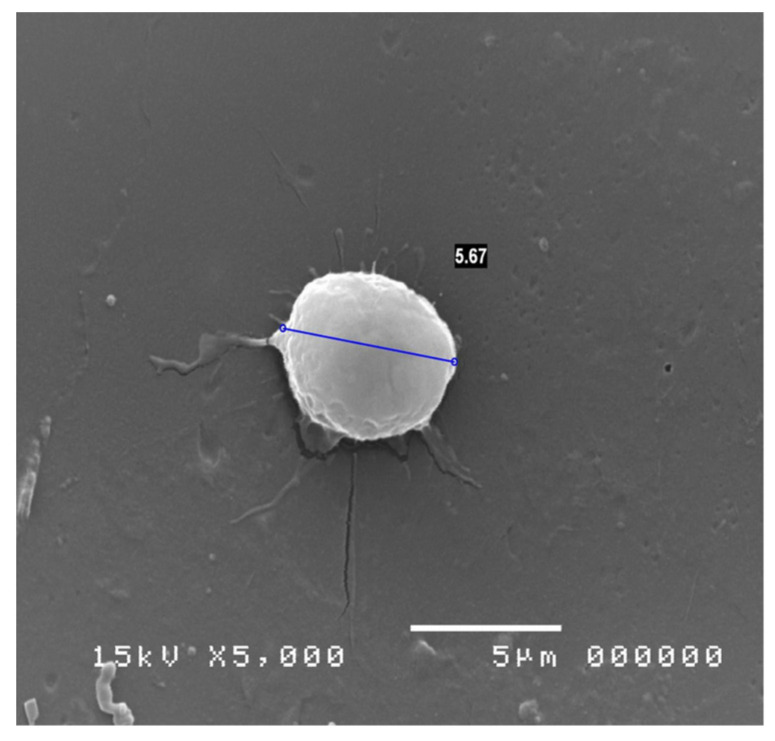
Acanthamoeba cyst at 5000×.

**Figure 6:**
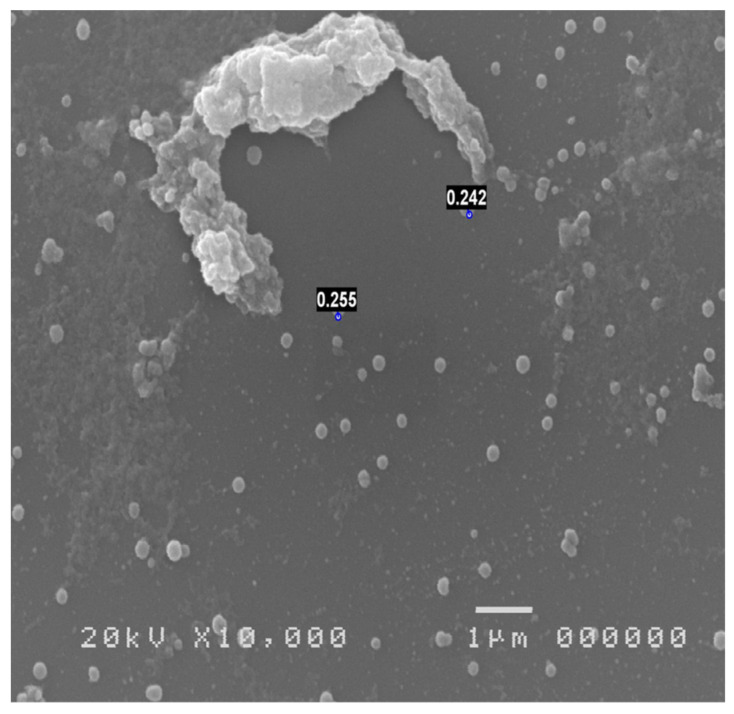
Chlamydia keratoconjunctivitis.

Other opportunities offered by SEM examination applied to impression cytology of the conjunctiva is the identification of inflammatory cells (Figure 4) and possible pathogens, including those, such as Acanthamoeba (Figure 5) and Chlamydia (Figure 6), which are not highlighted with normal culture tests and which may remain initially unknown in cases of an inflammatory process of uncertain origin [30,58].

**Figure 7:**
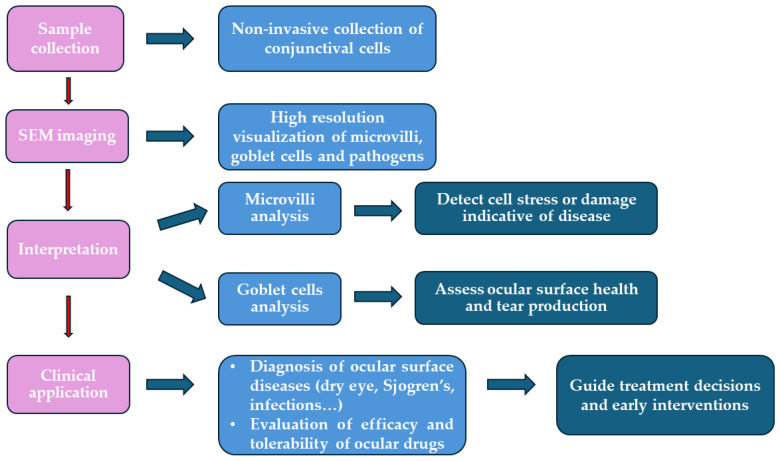
The flowchart reports the phases of scanning electron microscopy applied to impression cytology (ICSEM) with the clinical significance of each step.

The ICSEM process and its clinical relevance are summarized in the flow chart below (Figure 7).

## 5. Alternative Methods for Assessment of Microvilli 

Alternative methods proposed for conjunctival imaging are micro-Raman spectroscopy and in vivo confocal microscopy.

Some authors have proposed the use of micro-Raman analysis to reveal conjunctival microvillus abnormalities on specimens obtained by impression cytology from patients at different stages of dry eye syndrome. The experimental results demonstrate that Raman analysis, combined with the use of principal component analysis, can detect different stages of microvilli reduction. The results are promising for the use of Raman analysis for examination and comparable with those obtained through SEM evaluation [59].

The HRT II in vivo confocal microscope (IVCM) also allows for accurate study of both central and peripheral ocular surface epithelia compared to first-generation confocal microscopy devices. This technique highlights dramatic changes in the conjunctiva in ocular surface disease; in dry syndrome, in particular, squamous metaplasia, inflammatory cell infiltration, goblet cell depletion, and a nuclear snake-like chromatin pattern are found; in rosacea and in forms of limbal deficiency, corneal conjunctivalization (goblet cells together with conjunctival epithelial cells within the layer of corneal epithelium) has been observed. The images provided by this technique were in excellent correlation with the results obtained using impression cytology; however, they did not allow the researchers to appreciate early changes, such as those affecting the microvilli [60].

A clear comparison of these key techniques used in ocular surface evaluation, highlighting their specific uses, strengths, and limitations in clinical and research applications, is summarized in Table 2.

While SEM provides unparalleled three-dimensional imaging of microvilli morphology, it complements other non-invasive techniques like in vivo confocal microscopy (IVCM) and Raman spectroscopy. IVCM enables real-time imaging of conjunctival cells and tear film dynamics, making it practical for clinical use. Raman spectroscopy, on the other hand, focuses on biochemical changes, such as alterations in tear film proteins or lipids. Together, these methods can provide a holistic assessment of ocular surface health, bridging structural, cellular, and biochemical evaluations [7,60].

## 6. Discussion

The conjunctiva is a relatively little studied but incredibly important tissue, due to its key role in providing protection to the eye and maintaining the homeostasis of the ocular surface. Multiple diseases can compromise conjunctival function, resulting in serious consequences. Initial changes to the conjunctiva can be repaired with relative ease, but more serious conditions rely on surgeries and tissue grafts that generally do not provide adequate healing [61].

The morphological examination of microvilli is particularly valuable for an early diagnosis as it allows for the evaluation of initial alterations of the ocular surface in dry eye and other pathologic conditions; furthermore, with this test, it is possible to identify ultrastructural variations in response to toxic and inflammatory agents, as well as possible trophic effects of some substances. Damage to microvilli or their restoration may be indicative of the action of these products on ocular surface health [19].

Unlike more invasive biopsy methods and studies on cell lines, mainly reserved for scientific research, the ICSEM of the conjunctiva allows for an objective, effective, and non-invasive examination of the conjunctiva to be carried out in vivo in all cases of ocular pathologies linked to alterations of the microvilli. It is also possible to evaluate with this technique the effects of toxic substances and drugs on the ocular surface [55], collecting objective and reproducible morphological results, as demonstrated by the most recent studies.

The versatility of the method makes it suitable for both research and clinical practice purposes, for the early evaluation of different forms of epithelial distress of the ocular surface [55,56].

We believe that this diagnostic technique can be usefully applied in all cases of dry eye and persistent inflammation, for an evaluation of the actual conditions of the ocular surface and to set up a targeted and effective therapeutic strategy.

In addition, although not yet widely applied, SEM’s ability to provide high-resolution, three-dimensional images of conjunctival epithelium makes it a promising tool for early detection of ocular surface squamous neoplasia (OSSN); its capacity to identify cellular and subcellular morphological changes could complement existing techniques like in vivo confocal microscopy [61].

SEM could be extended to assess conjunctival changes due to UV exposure and degenerative conditions like pterygium, where it might focus on limbal epithelial stem-microenvironmental alteration, changes in microvilli density, epithelial cell morphology, and matrix alterations, providing insights into the pathophysiology and progression of these disorders [62].

SEM has proven highly sensitive in diagnosing conjunctival and corneal infections in patients with microbial keratoconjunctivitis who are unresponsive to broad-spectrum antibiotics. Its utility in identifying eosinophils, mast cells, and inflammatory changes on the conjunctival surface can aid in differentiating allergic from infectious conjunctival disorders, offering a critical advantage in guiding specific therapies [31,32].

Taking into account all these aspects, the ICSEM technique is a candidate for an elective technique for the diagnosis of ocular surface disorders, being able to highlight the effectiveness of the treatments carried out and the effect of drugs and pathological conditions on the state of microvilli with a greater sensitivity than common tests.

A comparative analysis with additional biomarkers, such as mucin expression (MUC5AC levels) and goblet cell density, could complement SEM findings [63,64,65]. These biomarkers provide insight into tear film quality and conjunctival health, offering a functional perspective alongside SEM’s structural analysis. Moreover, inflammatory markers like IL-6 and TNF-α could help identify early ocular surface inflammation, strengthening diagnostic accuracy [66,67,68,69].

Limitations of this technique are the requirement for specialized equipment (SEM), its cost, and the learning curve associated with having adequate competence to perform cytological evaluations.

Future research should focus on the development of standardized protocols and automated systems that can enhance its accessibility and application, overcoming the current limitations related to complex equipment and the need for highly skilled operators.

## Figures and Tables

**Table 1:** Classification of conjunctival microvilli according to scale from Del Prete et al. [55].

Grade 0	Grade 1	Grade 2	Grade 3	Grade 4
Microvilli on site	Microvilli on site	Microvilli on site	Microvilli on site	Smooth area for microvilli absence
Normal surface	Normal surface	Low alteration of the surface	High alteration of the surface	High alteration of the surface
High microvillar distribution	Low microvillar distribution	Microvillar distribution on spot	Microvillar sensible reduction with spotted smooth areas	Microvillar absence
Arborescent structure of microvilli	Structure of microvilli not totally arborescent	Pseudomicrovilli	Pseudomicrovilli	Smooth surface, moon-surface

**Table 2:** Comparative table summarizing the features, strengths, limitations, and clinical applications of different conjunctival imaging techniques including SEM, in vivo confocal microscopy, and Raman spectroscopy.

Techniques	Resolution and Imaging	Strengths	Limitations	Clinical Applications
Scanning Electron Microscopy (SEM)	High-resolution, 3D imaging of microvilli and epithelial cell surfaces. Can assess microvilli alterations in detail.	Provides detailed visualization of conjunctival microvilli, detects early structural damage. Ideal for research purposes.	Expensive, time-consuming for sample preparation, not dynamic, cannot image in vivo. Not easily accessible in clinics.	Monitoring dry eye disease, assessing drug-induced damage, studying microbial keratitis and tear substitutes’ efficacy.
In Vivo Confocal Microscopy (IVCM)	Moderate resolution, real-time imaging of conjunctival and corneal epithelial cells, allowing for dynamic evaluation.	Non-invasive, real-time, allows for monitoring disease progression or treatment effects dynamically.	Lower resolution than SEM, limited visualization of microvilli, may miss subtle structural changes.	Assessing dry eye, rosacea, ocular surface inflammation, tracking epithelial health, evaluating corneal nerve density and morphology, identifying bacterial, fungal, and Acanthamoeba keratitis by visualizing inflammatory cells and infectious organisms.
Raman Spectroscopy	Molecular analysis of tissues by detecting vibrations of molecular bonds. Lower spatial resolution compared to SEM.	Non-invasive, can detect molecular and biochemical changes. Useful for detecting early-stage disease.	Does not provide detailed structural images like SEM; complex analysis is needed for interpretation.	Detecting molecular changes in dry eye disease, early diagnosis of ocular surface abnormalities.

## Data Availability

All data are provided in the main text.
